# Novel Porcine Retina Cultivation Techniques Provide Improved Photoreceptor Preservation

**DOI:** 10.3389/fnins.2020.556700

**Published:** 2020-10-06

**Authors:** Natalie Wagner, Sabrina Reinehr, Maurice R. Gammel, Andrea Greulich, José Hurst, H. Burkhard Dick, Sven Schnichels, Stephanie C. Joachim

**Affiliations:** ^1^Experimental Eye Research Institute, University Eye Hospital, Ruhr-University Bochum, Bochum, Germany; ^2^University Eye Hospital, Centre for Ophthalmology, Tübingen, Germany

**Keywords:** age-related macular degeneration, porcine, photoreceptor, optical coherence tomography, organotypic retina culture, opsin, rhodopsin

## Abstract

Age-related macular degeneration (AMD) is the leading cause of blindness in industrialized countries among people over 60 years. It has multiple triggers and risk factors, but despite intense research efforts, its pathomechanisms are currently not completely understood. AMD pathogenesis is characterized by soft drusen in Bruch’s membrane and involves the retinal pigment epithelium–Bruch’s membrane-choroid complex and adjacent structures, like photoreceptors. This study explores the potential of novel cultivation techniques to preserve photoreceptors in retinal explants to gain better insights in AMD pathology. The porcine retina explants were cultured for 4 and 8 days using three different explantation techniques, namely, control (photoreceptors facing down, touching the filter), filter (photoreceptors facing up, turned sample using a filter), and tweezers (photoreceptors facing up, turned sample using tweezers). Optical coherence tomography revealed that the tweezers method had the best capacity to limit thinning of the retinal explants. Both novel methods displayed advantages in maintaining outer segment thickness. Additionally, immunofluorescence evaluation revealed a better preservation of opsin^+^ cells and rhodopsin signal intensity in both novel methods, especially the tweezers method. Furthermore, RT-qPCR analysis demonstrated an upregulation of *OPSIN* and *RHODOPSIN* mRNA expression in tweezers samples at 8 days. Amacrine and bipolar cell numbers were not altered at day 4 of cultivation, while cultivation until 8 days led to reduced bipolar cell numbers. At 4 days, *CALRETININ* mRNA was upregulated in filter samples, but *protein kinase C alpha* expression was downregulated. Retinal ganglion cells were diminished in both novel techniques due to a direct physical contact with the insert. Remarkably, no difference in *TUBB3* mRNA expression was detected among the techniques. Nevertheless, both novel methods exhibited an improved retention of photoreceptor cells. In conclusion, the tweezers technique was the most promising one. Due to the high homology of the porcine to the human retina, it provides a reasonable alternative to *in vivo* rodent models. Consequently, an adapted coculture system based on the current findings may serve as an *ex vivo* model suitable to analyze AMD pathomechanisms and novel therapeutic approaches.

## Introduction

Vision loss is one of the most dreaded constraints together with cancer and Morbus Alzheimer (Scott et al., 2016). One of the leading causes of blindness in industrialized countries, among people over the age of 60, is age-related macular degeneration (AMD) (Klein et al., 1992, 2004; Nowak, 2006; Wong et al., 2014). The early form of AMD is characterized by the presence of lipid-rich deposits, e.g. drusen, and retinal pigment epithelium (RPE) hypopigmentation and hyperpigmentation (Curcio et al., 2013; Klettner et al., 2013). Drusen are located beneath the RPE and consist of many components, such as lipids, amyloid proteins, immune complexes, and complement proteins (Mullins et al., 2000; Crabb et al., 2002). The atrophic (dry) late form is characterized by areas of RPE and photoreceptor degeneration, so-called geographic atrophy. The exudative (wet) form has choroidal neovascularization, resulting in edema and photoreceptor degeneration (Ferris et al., 2013). Several risk factors, such as advanced age, genetic disposition, family history of AMD, race, smoking, obesity, or hypertension, are known to be involved in this multifactorial disease (Mares et al., 2011; Grassmann et al., 2015; Merle et al., 2019). In AMD, characteristic extracellular lipid-rich deposits between outer retinal cells are formed (Buitendijk et al., 2013). RPE cells accumulate lipofuscin, which is a remnant of retinoid metabolites from shed photoreceptor outer-segment membranes (Eldred et al., 1982). The precise role of lipofuscin in AMD is currently under investigation (Fritsche et al., 2014; Gambril et al., 2019; Bermond et al., 2020). Overall, the exact AMD pathogenesis is still not fully understood.

Appropriate *in vivo* models for this retinal disease are limited. In most animal models, the disease induced is acute and the animals are specially bred and killed for the experiment. There is a need for reliable, reproducible, and close-to-human *ex vivo* models, which could be an alternative to animal, especially rodent, models (Dithmar et al., 2000; Shah et al., 2015; Carver et al., 2017; Park et al., 2017; Tode et al., 2018). These rodent models are either based on laser-induced injuries to the RPE and Bruch’s membrane or AMD-like defects, which are caused by genetic knockouts. Additionally, like most animals, rodents lack a macula (Huber et al., 2010). Moreover, they have different photoreceptor types. In particular, they only have two types of cones, while humans have three types enabling red light vision (Jacobs et al., 2001). The porcine eye resembles the human eye much closer in regard to anatomy and morphology. Hence, they are often used as *ex vivo* animal models in ophthalmologic research (Schnichels et al., 2020). Especially, the structure of the retinal layers is quite comparable to the human one due to similar development (Gu et al., 2007). However, porcine eyes do not have a macula with a fovea but a comparable central zone called visual streak (Chandler et al., 1999; Hendrickson and Hicks, 2002; Kiilgaard et al., 2007; Bertschinger et al., 2008). The broad horizontal visual streak is located in the tapetal region slightly superior and temporal to the optic nerve and contains the greatest density of photoreceptors and retinal ganglion cells (RGCs) (Maggs et al., 2008). For example, cones can be found in a density of about 15,000 to 40,000 cells/mm^2^ (Nicoli et al., 2009), similar to the human macula (Bertschinger et al., 2008). Besides that, porcine eyes can be easily obtained from abattoirs, as a side product of the food industry. Hence, these animals are not solely bred and killed for research experiments.

Our study aimed to investigate a novel *ex vivo* porcine organ culture model where photoreceptors are well preserved. The analysis of photoreceptor degeneration processes is of crucial importance when composing an *ex vivo* AMD model. Our novel tweezers method provides a good preservation of photoreceptor outer segments; thus, it could be used in future studies as part of an *ex vivo* AMD model.

## Materials and Methods

### Preparation and Cultivation of Porcine Neuroretina Explants

**FIGURE 1:**
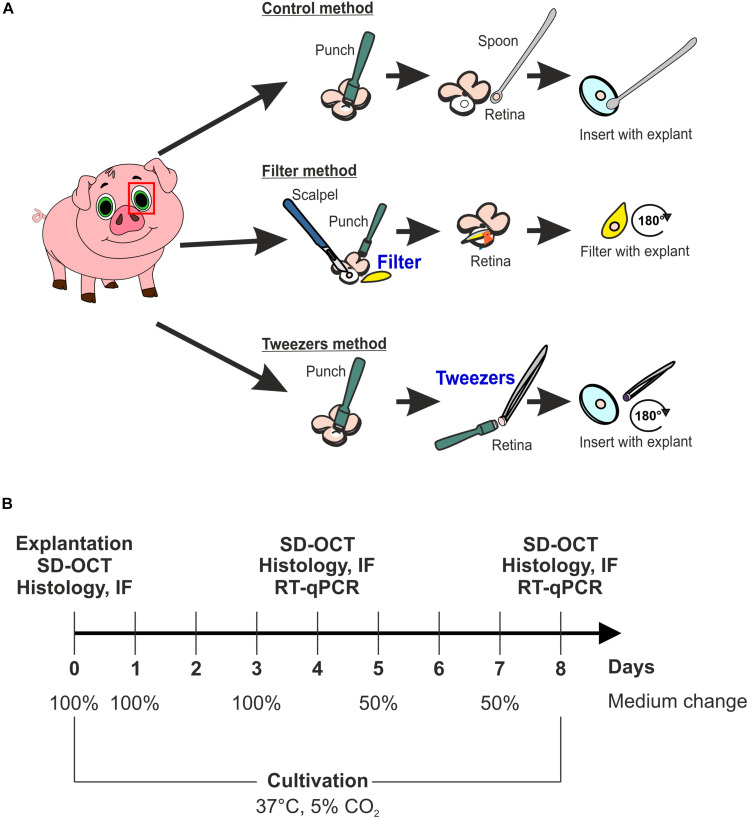
Scheme of the used explant methods and study design. **(A)** Scheme of the three different explantation techniques, named control, filter, and tweezers method. The fourth group was a native one consisting of samples gained *via* the three different methods, which were analyzed at day 0. **(B)** Timeline of the study to investigate which explantation method best preserves the photoreceptor morphology *ex vivo*. Three techniques were compared during the cultivation periods of 4 and 8 days using spectral domain optical coherence tomography (SD-OCT), immunofluorescence (IF), and quantitative real-time PCR (RT-qPCR). Native samples were also included in the analysis.

Porcine eyes were obtained from the local abattoir and immediately transported to the laboratory, while stored on ice. The eyes were processed within 3 h after animals were sacrificed. First, eyes were cleaned by removing excessive tissue with scissors and immersed in 70% ethanol. Subsequently, they were dissected with a scalpel under a laminar flow hood, and an incision in the cornea was made. Then, cornea, lens, and vitreous were discarded, and the eye cup was washed in sterile phosphate buffered saline (PBS) to eliminate vitreous body residues. To protect the photosensitive retina, the posterior eyeball was rinsed with medium (Neurobasal-A medium, Life Technologies, Carlsbad, CA, United States) supplemented with 0.8 mM L-glutamine (Life Technologies), 2% B27 (Life Technologies), 1% N2 (Life Technologies), and 2% penicillin/streptomycin (Sigma-Aldrich, St. Louis, MO, United States). A cloverleaf-like structure was generated to gain retinal explant from the visual streak. Next, three different techniques, named control, filter, and tweezers method, were performed to obtain retina explants using a dermal punch (∅ = 6 mm, Pmf medical AG, Cologne, Germany). In the control method, explants were obtained by punching out retinal samples. Then, the RPE was removed by washing retinal explants in Neurobasal-A medium. Finally, retinal samples were placed on a Millicell culture insert (Millipore, Burlington, VT, United States) with the ganglion cell layer (GCL) facing up (Kuehn et al., 2016, 2017; Figure 1A). The filter technique was adapted from Wang et al. (2011) (Figure 1A). Here, a punch was made through the retina, and then a sterile filter paper was carefully applied onto the stamped-out retina sample (Wang et al., 2011). Following, the explant was slowly lifted, the GCL attached to the filter, and placed in a six-well plate (Millipore). The third technique was also performed using a dermal punch. However, much more pressure was exerted to gain an explant from the neuroretina and the underlying structures including the sclera. Subsequently, the sample was lifted with tweezers and rotated 180 degrees. Afterward, the explant was placed on a cell culture insert. After this step, the sclera and underlying structures, like choroid and RPE, were removed with tweezers, pinching the sclera, to leave the neuroretina explant on the insert (Figure 1A). To have an adequate uncultivated control, samples of the three different methods at day 0 were used as native controls. Finally, retinal samples were cultured in 1 ml medium at 37°C and 5% CO_2_ for 4 and 8 days. The medium was completely replaced on days 0, 1, 2, and 3. At days 5 and 7, only 50% of the medium was exchanged. At days 4 and 8, the retinal samples were obtained for spectral domain optical coherence tomography (SD-OCT, *n* = 5/group), quantitative real-time PCR (RT-qPCR, *n* = 5/group), and histological or immunofluorescence (IF) analysis (*n* = 9–10/group; Figure 1B).

In total, four different groups were compared. Samples that were obtained at day 0 using control, filter, and tweezers technique comprised the native group (Supplementary Figure S1). The second group of retinas with GCL facing up was the control technique. The third group consisted of retinas extracted by the filter technique, photoreceptors facing up. The fourth group consisted of retinas obtained by the tweezers method, photoreceptors facing up.

### Optical Coherence Tomography

The high-resolution OCT examination of porcine retina samples was performed with an SD-OCT (Spectralis, Heidelberg Engineering, Heidelberg, Germany). For the exploration of the explants, a customized mounting device was used (Schnichels et al., 2016). The holder was adapted to fit the 12-mm ∅ cell culture inserts. The retina samples of all groups (*n* = 5/group) were investigated immediately after preparation at day 0 (= native) and after 4 and 8 days. Three 30°-line scans (ART:100) and an additional group scan, consisting of 20 frames, were performed. During the whole procedure, attention was paid to keep constant aseptic conditions and to prevent a dehydration of the explant. The retina thickness was evaluated according to established protocols (Schnichels et al., 2016; Klemm et al., 2019). To this end, the thickness was measured five times per picture *via* ImageJ (version 1.3u, National Institutes of Health, Bethesda, MD, United States). Three pictures per explant were taken, and a mean of 15 values was calculated per sample.

### Preparation of Retinal Sections for (Immuno)Histology

In order to cut cross sections of the retina samples, they were fixed with 4% paraformaldehyde (PFA; Merck, Darmstadt, Germany) for 15 min. Afterwards, the explants were drained with 15% sucrose solution (Sigma-Aldrich) for 15 min and 30% sucrose solution for 30 min. Finally, the explants were embedded in NEG-50 Tissue Tek medium (Thermo Fisher Scientific, Waltham, MA, United States) and stored at −80°C. Subsequently, a microtome (Thermo Fisher Scientific) was used to prepare 10 μm cross sections. Three tissue sections were placed on a Histobond slide (Paul Marienfeld GmbH & Co., KG, Lauda-Königshofen, Germany) and air-dried at room temperature overnight. For histological analyses, all slides were fixed in ice-cold acetone for 10 min on the following day and stored at −80°C.

## Hematoxylin and Eosin Staining and Immunofluorescence of Retinal Cross Sections

To evaluate morphologic changes, hematoxylin and eosin (H&E; Merck) stains were performed (Fischer et al., 2008). Thereby, nuclei are stained blue, whereas the cytoplasm and extracellular matrix appear pink. Two pictures of the central region of each cross section (six sections per sample) were taken *via* a microscope equipped with a CCD camera (Axio Imager M1, Zeiss, Oberkochen, Germany) at 200 × magnification. Afterward, the retinal thickness was measured with a measurement tool using Zen software (Zeiss). Per picture, the total thickness as well as the outer (OS) and inner segment (IS) thickness (= bacillary layer) was measured at three positions of the retina. For the total retinal thickness, the measurement tool was used to scale the distance between the GCL and the outer segments of the photoreceptor cells. To evaluate the bacillary layer (OS and IS), we measured the outermost layer of the retina from the outer nuclear layer to the outer segment of the photoreceptor cells. The average values of all three methods at 0 days were classified as the native group. The whole retina and bacillary layer thickness of the native group was defined as 100%.

To identify different cell types of the retina, specific primary antibodies (Table 1) were used for IF staining (Kuehn et al., 2016; Hurst et al., 2017). First, retinal sections were defrosted and dried at 37°C for at least 15 min. Then, they were rinsed in PBS (Biochrome, Schaffhausen, Switzerland) and blocked with antisera (goat or donkey) diluted in 0.1–0.2% Triton X-100 (Sigma-Aldrich) in PBS (PBST) and 1% bovine serum albumin. Thereafter, sections were incubated with primary antibodies (Table 1) containing antisera solution diluted in PBST at room temperature overnight. Next, the slides were incubated with secondary antibodies (Table 1), which were labeled with Alexa Fluor 488 or Alexa Fluor 555, at room temperature for 1 h. Subsequently, nuclei were counterstained with 4′,6-diamidino-2-phenylindole (DAPI; 0.01 μg/ml; Serva Electrophoresis, Heidelberg, Germany). Slides, where the primary antibody solution was omitted, served as negative controls. At the last step, all slides were covered in Shandon mount media (Thermo Fisher Scientific).

**TABLE 1:** List of primary and secondary antibodies used for immunofluorescence staining.

**Primary antibodies**			**Secondary antibodies**		
**Antibody**	**Company**	**Dilution**	**Antibody**	**Company**	**Dilution**
Anti-calretinin	Santa Cruz Biotechnology	1:100	Donkey anti-goat Alexa Fluor 488	Dianova	1:500
Anti-opsin	Merck Millipore	1:1,200	Donkey anti-rabbit Alexa Fluor 555	Invitrogen	1:500
Anti-PKCα	Santa Cruz Biotechnology	1:300	Goat anti-mouse Alexa Fluor 488	Invitrogen	1:500
Anti-RBPMS	Merck Millipore	1:400	Donkey anti-rabbit Alexa Fluor 555	Invitrogen	1:500
Anti-rhodopsin	Abcam	1:400	Goat anti-mouse Alexa Fluor 488	Invitrogen	1:500

*PKCα, protein kinase C alpha; RBPMS, RNA-binding protein with multiple splicing.*

To evaluate the retinal IF pictures, four images per section were taken using an Axio Imager M1 or M2 microscope (Zeiss). For further evaluation, images were masked using Ant Renamer 2 software (version 2.10, Antoine Potten, Brussels, Belgium) and then cut in predefined sections (800 × 600 pixels) with Corel PaintShop Pro X8 (Corel, Corel Corporation, Ottawa, ON, Canada). Within those predefined windows, calretinin-, opsin-, protein kinase C alpha (PKCα)-, and RNA-binding protein with multiple splicing (RBPMS)-positive labeled cell bodies were counted using the ImageJ plugin “cell counter.” For the signal intensity analysis of rhodopsin, ImageJ was used and all the images were transformed into gray scale. In the next step, the background was subtracted (50 pixels) and a lower and upper threshold (lower: 11.55, upper: 82.39) was determined to quantify the rhodopsin signal intensity per section (Reinehr et al., 2016).

### Quantitative Real-Time PCR

RNA isolation and cDNA synthesis of porcine retina explants were performed as described previously (Hurst et al., 2017) and according to the manufacturer’s instructions with a MultiMACS cDNA Kit (Miltenyi Biotec, Bergisch Gladbach, Germany). For specific primer design, Primer3 software, based on the published GenBank sequence (GenBank: *sus scrofa* taxid:9823)^1^, was used (Table 2). RT-qPCR was carried out (CfX 96 System, Bio-Rad Laboratories, Inc., Hercules, CA, United States) using the SYBR Green SsoAdvanced^TM^ Universal SYBR^®^ Mastermix (Bio-Rad Laboratories). In a reaction volume of 20 μl, 5 ng of cDNA were present. Final primer concentration was 2 μM, and samples were analyzed twice. The relative expression of the target genes in the novel groups filter and tweezers in comparison to the control group was calculated with REST© 2009 (Qiagen, Hilden, Germany) and expressed as the fold changes in gene expression. The expression levels of the target genes were normalized against the housekeeping genes *ACTB* (β*-ACTIN*) and *RPL4* (*ribosomal protein L4*) (Wang et al., 2014).

**TABLE 2:** List of quantitative real-time PCR (RT-qPCR) primer pairs used. *ACTB* and *RPL4* served as housekeeping genes.

**Gene**	**Oligonucleotides 5′→ 3′**	**GenBank accession number**	**Amplicon size**
*ACTB for*	ctcttccagccttccttc	XM_021086047.1	178
*ACTB rev*	gggcagtgatctctttct		
*CALBINDIN 2 for*	tgaacccaagctccaagagt	NM_001194980.2	176
*CALBINDIN 2 rev*	aaaaggtgaagatggcgttg		
*OPSINM for*	ggggagcatcttcacctaca	NM_001011506.1	244
*OPSINM rev*	gatgatggtctctgccaggt		
*PKC*α *for*	accgaacaacaaggaacgac	XM_021066740.1	163
*PKC*α *rev*	ctgagctccacgtttccttc		
*RHODOPSIN for*	tccaggtacatcccagaagg	NM_214221.1	151
*RHODOPSIN rev*	gctgcccatagcagaagaag		
*RPL4 for*	caagagtaactacaaccttc	XM_005659862.3	122
*RLP4 rev*	gaactctacgatgaatcttc		
*TUBB3 for*	cagatgttcgatgccaagaa	NM_001044612.1	164
*TUBB3 rev*	gggatccactccacgaagta		

*for, forward; rev, reverse.*

### Statistical Analyses

The SD-OCT, histology, and IF results are presented as mean ± SEM, while RT-qPCR results are displayed as median ± quartile + minimum/maximum. A *p*-value < 0.05 was considered statistically significant. The level of significance was defined as ^∗^*p* < 0.05, ^∗∗^*p* < 0.01, and ^∗∗∗^*p* < 0.001 when compared to the native group, ^#^*p* < 0.05, ^##^*p* < 0.01, and ^###^*p* < 0.001 when compared to the control group, and ^¥^*p* < 0.05 and ^¥¥^*p* < 0.01 when compared to the filter group.

For the evaluation of SD-OCT, histology, and IF data, groups were compared by ANOVA, followed by Tukey *post hoc* test (Statistica; version 13.3; Dell Software, Round Rock, TX, United States). The CT values of the RT-qPCR analysis were evaluated with REST© 2009 software (Qiagen).

## Results

### Preservation of Retinal Thickness in Tweezers Samples

**FIGURE 2:**
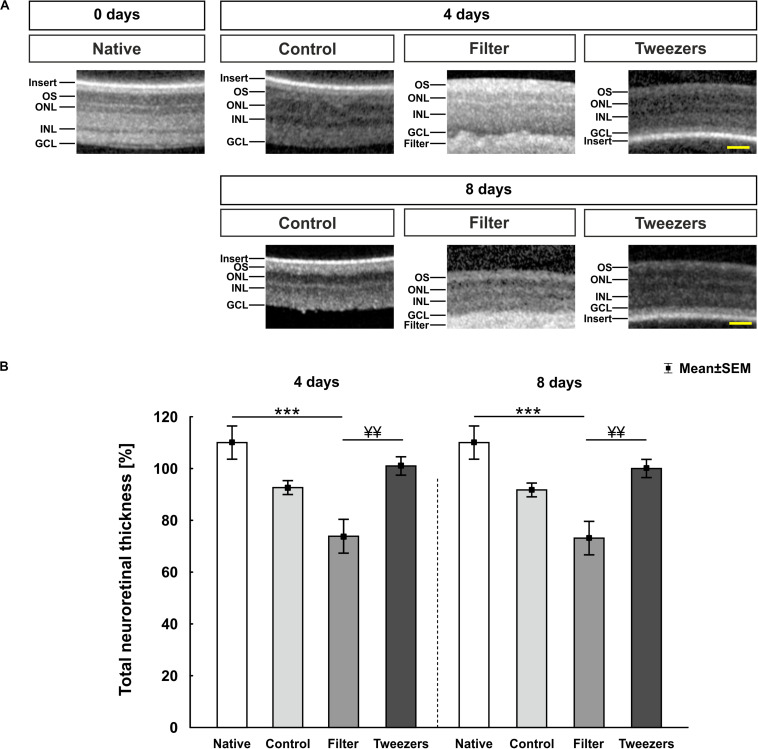
Spectral Domain Optical Coherence Tomography (SD-OCT) analysis of porcine retinas. **(A)** Exemplary pictures of all measured time points and used techniques. Samples of all three explantation methods and the native group were investigated at 0 (= native), 4, and 8 days *via* SD-OCT. **(B)** At 4 days, no changes were noted in regard to the retinal thickness between control and native samples. A significantly thinner retinal thickness was revealed in filter (*p* < 0.001), but not in tweezers samples, compared to native retinas. No alterations were noted in filter and tweezers retinas compared to the controls. The filter group showed a significantly decreased retinal thickness compared to the tweezers group (*p* = 0.008). The retinal thickness of control and native samples was comparable at 8 days. While a significant thinning of the filter group was observed compared to native retinas (*p* < 0.001), no changes were noted between tweezers and native samples. Furthermore, the retinal thickness of the filter group was significantly diminished compared to tweezers samples (*p* = 0.008). OS, photoreceptor outer segments; ONL, outer nuclear layer; OPL, outer plexiform layer; INL, inner nuclear layer; IPL, inner plexiform layer; GCL, ganglion cell layer. Scale bars: 50 μm, values are mean ± SEM. *n* = 5/group. ***p < 0.001 vs. native group; ^¥¥^*p* < 0.01 vs. filter group.

The SD-OCT enabled an assessment of porcine retina samples during different time points. During all investigated points in time (zero = native, 4 and 8 days), a detailed observation of the layers was possible (Figure 2A). The filter paper/insert could be clearly identified above (native, control) or below (filter, tweezers) the explants *via* SD-OCT. The measurement of the retinal thickness revealed no changes in control samples compared to native ones at 4 days (*p* = 0.11; Figure 2B). In the filter group, a significantly decreased retina thickness could be noted compared to native samples (*p* < 0.001), while no differences were observed between tweezers and native retinas (*p* = 0.60). No differences were revealed when comparing filter (*p* = 0.08) and tweezers samples (*p* = 0.66) to the control group. A better preservation of the retinal thickness was observed in tweezers samples compared to filter retinas at 4 days (*p* = 0.008). At 8 days of cultivation, the retinal thickness in the control group did not differ from native retinas (*p* = 0.08). The retinal thickness in the filter group was significantly diminished compared to native samples (*p* < 0.001). The tweezers samples showed a similar thickness in comparison to native ones (*p* = 0.52). Both novel methods, filter (*p* = 0.08) and tweezers (*p* = 0.66), showed no differences in the retinal thickness compared to control samples. Eight days after cultivation, the retinal thickness in tweezers samples was significantly higher compared to filter retinas (*p* = 0.008).

### Less Reduction in the Total and Photoreceptor Layer Thickness Using the Novel Methods

**FIGURE 3:**
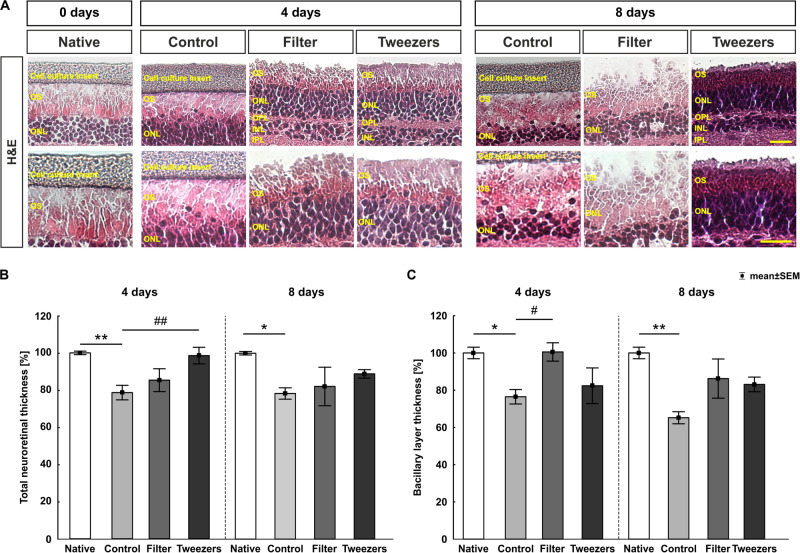
Total and bacillary layer thickness measurement in stained retinas. **(A)** Representative images of H&E-stained retinas for all three explantation methods and the native group in 400 × (upper panel) and 630 × magnification (lower panel). **(B)** At 4 days, a significant reduction of the total retinal thickness in control compared to native samples (*p* = 0.005) was observed, while a preservation was detected in the novel techniques filter and tweezers when compared to native samples. Comparing tweezers to control samples, a significantly thicker retinal thickness was noted (*p* = 0.009), while a similar thickness was observed between filter and control retinas. Similar results were found at 8 days. A reduction of the total retinal thickness was revealed comparing control to native samples (*p* = 0.04). A conservation of the total retinal thickness was detected comparing filter and tweezers to native or control retinas. **(C)** A significant thinning of the bacillary layer was seen in the control group compared to native (*p* = 0.04) and filter samples (*p* = 0.046). However, a better-preserved bacillary layer was noted in the tweezers method compared to the native and control samples at 4 days. A reduction in the bacillary layer thickness was measured at day 8 comparing the control and native samples (*p* = 0.001). The novel methods filter and tweezers maintained the bacillary layer thickness better than did control retinas. OS, photoreceptor outer segments; ONL, outer nuclear layer; OPL, outer plexiform layer; INL, inner nuclear layer; IPL, inner plexiform layer. Scale bars: 20 μm, values are mean ± SEM. *n* = 9–10/group. *p < 0.05 and **p < 0.01 vs. native group; ^#^*p* < 0.05 and ^##^*p* < 0.01 vs. controls.

Hematoxylin and eosin-stained porcine retinas enabled to distinguish between nuclear and cytoplasmic structures, in particular measuring the thickness of the total retina from GCL to the outer photoreceptor segments. The bacillary layer measured spanning from the outer nuclear layer until the outer segment of the photoreceptors (Figure 3A).

When comparing retinas of the control group to native samples cultivated for 4 days, a significant reduction of the total retinal thickness was observed (*p* = 0.005; Figure 3B). Comparing filter (*p* = 0.10) and tweezers (*p* = 0.99) to the native samples, no significant differences were seen. When filter and control samples were compared, no differences were detected (*p* = 0.70). A significantly better preservation of the retinal thickness was noted in tweezers samples compared to the controls at 4 days (*p* = 0.009). Similar effects could be observed when the retinal explants were cultivated for 8 days. At this time point, there was a significant decrease in the retina thickness in the control group compared to native retinas (*p* = 0.04). Interestingly, a good preservation of the total retina thickness could be achieved by the filter (*p* = 0.12) and tweezers method (*p* = 0.50) when compared to native samples after 8 days of cultivation. Also, no changes were noted when comparing filter (*p* = 0.96) and tweezers retinas (*p* = 0.54) to controls.

Going into detail, by assessing only the bacillary layer thickness at 4 days (Figure 3C), a significant reduction was detected in controls compared to native samples (*p* = 0.04). Comparing filter (*p* = 1.0) and tweezers bacillary layer (*p* = 0.17) to native samples, no differences were noted. A better preservation of this layer was visible in filter retinas when compared to control samples (*p* = 0.046), while no differences were noted between tweezers and control samples (*p* = 0.89). After 8 days of cultivation, a significant reduction of the bacillary layer was also measurable in control compared to native samples (*p* = 0.001). On the other hand, a well-preserved bacillary layer was observed in filter (*p* = 0.39) and tweezers methods (*p* = 0.22) when compared to native samples. Filter (*p* = 0.08) and tweezers bacillary layer (*p* = 0.18) were similar to controls.

### Better Survival of Rods and L-Cones With the Novel Methods

**FIGURE 4:**
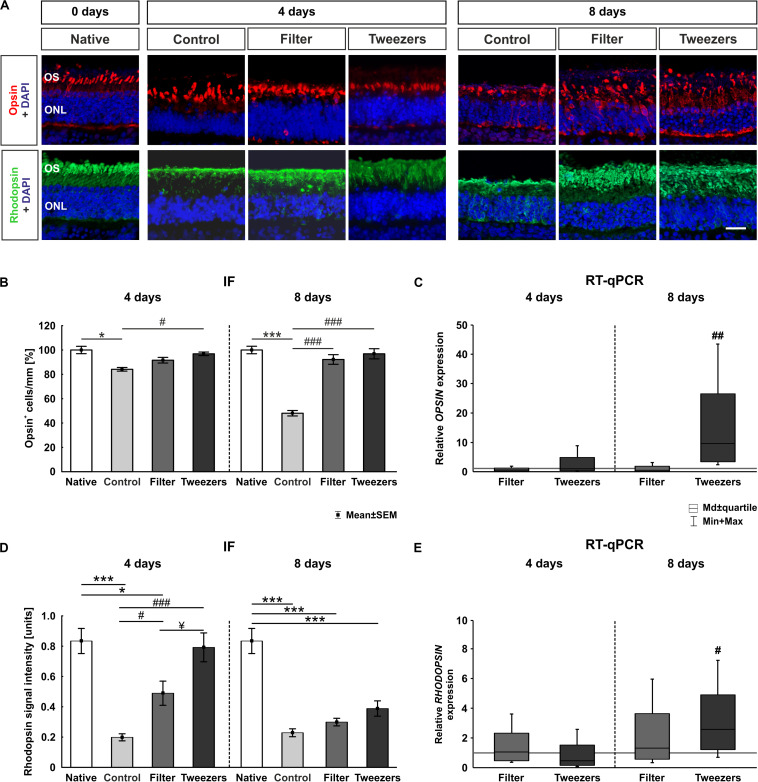
Analysis of photoreceptors. **(A)** Exemplary immunofluorescence pictures of opsin (red) staining for L-cones and rhodopsin (green) staining for rods in photoreceptor outer segments. Nuclei were labeled with 4′,6-diamidino-2-phenylindole (DAPI) (blue). **(B)** Fewer opsin^+^ cells were found in control compared to native retinas (*p* = 0.02). The filter and tweezers method showed, compared to native retinas, a better preservation over a cultivation period of 4 days. The number of opsin^+^ cells was significantly higher in tweezers samples (*p* = 0.04) compared to the control ones, while no changes were noted between filter and control retinas. A severe loss of opsin^+^ cells was discovered in the control compared to native samples after 8 days (*p* < 0.001). In the novel methods filter and tweezers, the number of opsin-labeled cells was comparable to native retinas. When comparing filter and tweezers samples (both: *p* < 0.001) to the controls, more opsin^+^ cells could be detected. **(C)** *OPSIN* expression was not altered at 4 days. *OPSIN* mRNA expression in tweezers samples was significantly upregulated compared to control retinas at 8 days (*p* = 0.002). **(D)** The rhodopsin signal found in control (*p* < 0.001) and filter samples (*p* = 0.01) was significantly less intense at 4 days compared to that in native samples. A significantly higher signal intensity was documented in tweezers (*p* < 0.001) and filter retinas (*p* = 0.047) compared to the controls. Moreover, a higher rhodopsin signal intensity was observed in tweezers retinas compared to filter samples (*p* = 0.04) at 4 days. At 8 days of cultivation, all three methods showed a significantly diminished rhodopsin intensity compared to native samples (all: *p* < 0.001). **(E)** *RHODOPSIN* mRNA expression was not altered at 4 days. Quantitative real-time PCR (RT-qPCR) examination of *RHODOPSIN* demonstrated an upregulation in tweezers samples compared to control retinas at 8 days (*p* = 0.02). OS, photoreceptor outer segments; ONL, outer nuclear layer. Scale bar: 20 μm, values are mean ± SEM for immunofluorescence (IF) and median ± quartile + min/max for RT-qPCR. IF: *n* = 9–10/group; RT-qPCR: *n* = 5/group. *p < 0.05 and ***p < 0.001 vs. native group; ^#^*p* < 0.05, ^##^*p* < 0.01, and ^###^*p* < 0.001 vs. controls; ^¥^*p* < 0.05 vs. filter group.

Porcine retinal cross sections of all three methods and corresponding native controls were stained with opsin to mark L-cones and with rhodopsin to label rods (Figure 4A). The native explants had an almost intact photoreceptor morphology and structure. L-cones were found organized in orderly rows, and rods appeared in organized laminar structures. No striking differences were observed in the organization of the L-cones, located in the outer photoreceptor segment, comparing native, filter, and tweezers samples after 4 days, while the opsin^+^ and rhodopsin^+^ cells in the control group looked different. In detail, the opsin^+^ L-cone cells appeared more disorganized, and the rhodopsin^+^ area seemed thinner, rather atrophic. Eight days after cultivation, the opsin^+^ cells appeared to be more disorganized in all three techniques compared to native samples.

At 4 days, a loss of L-cones was noted in the control group compared to native samples (*p* = 0.02; Figure 4B). There was no significant loss of opsin^+^ cones in retinas gained *via* filter (*p* = 0.62) and tweezers technique (*p* = 0.97) when compared to native samples. While no changes could be observed in filter retinas (*p* = 0.41), the number of opsin^+^ cells was significantly higher in tweezers samples than in control ones at 4 days (*p* = 0.04). A severe loss of opsin^+^ cells was discovered after 8 days of cultivation in control compared to native samples (*p* < 0.001). The number of L-cones was comparable in filter (*p* = 0.62) and tweezers samples (*p* = 0.97) compared to native ones. Significantly more opsin^+^ cells were detected in the two novel methods (filter: *p* < 0.001; tweezers: *p* < 0.001) compared to control retinas.

No differences were identified when comparing the *OPSINM* mRNA expression in both novel methods to controls at 4 days (tweezers: 0.94-fold, *p* = 0.9; filter: 0.6-fold, *p* = 0.3; Figure 4C). Accordingly, no differences in *OPSINM* expression were seen when the filter method was compared to the controls at 8 days (0.5-fold, *p* = 0.3). Interestingly, an upregulation in *OPSINM* mRNA expression was demonstrated in tweezers samples compared to the controls at 8 days of cultivation (9.6-fold, *p* = 0.002).

Additionally, the signal intensity of rhodopsin was evaluated (Figure 4A). At 4 days, the signal intensity of control (*p* < 0.001) and filter retinas (*p* = 0.013) was significantly lower than in native samples (Figure 4D). Tweezers and native samples, on the other hand, showed nearly identical intensities (*p* = 0.98). The signal intensity of rhodopsin was significantly higher in tweezers (*p* < 0.001) and filter samples (*p* = 0.047) compared to the controls. When comparing both novel groups, the rhodopsin intensity was significantly higher in tweezers samples compared to filter retinas (*p* = 0.04). After 8 days in cultivation, a clearly diminished rhodopsin signal intensity was documented in all three groups compared to native samples (control: *p* < 0.001, filter: *p* < 0.001, tweezers: *p* < 0.001). No difference was observed when comparing filter (*p* = 0.88) and tweezers samples (*p* = 0.07) to control retinas.

*RHODOPSIN* mRNA expression was not altered in filter (1.1-fold, *p* = 0.85) and tweezers samples (0.5-fold, *p* = 0.22) compared to the controls at 4 days of cultivation (Figure 4E). *RHODOPSIN* mRNA expression in filter samples was not significantly altered at 8 days (1.3-fold, *p* = 0.56). However, we discovered a significant upregulation of *RHODOPSIN* mRNA expression in tweezers samples (2.6-fold, *p* = 0.02) in comparison to control samples.

### Comparable Amacrine Cell Numbers but Loss of Bipolar Cells at 8 Days

**FIGURE 5:**
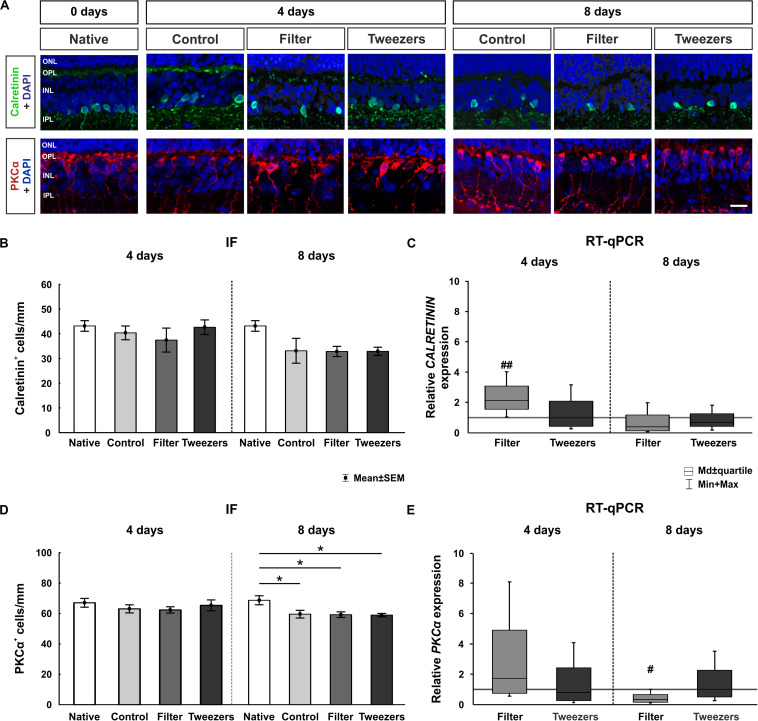
Effect on cells in the inner nuclear layer. **(A)** Amacrine cells were labeled with calretinin (green) and bipolar cells with protein kinase C alpha (PKCα) (red). Cell nuclei were stained with 4′,6-diamidino-2-phenylindole (DAPI) (blue). **(B)** No differences were observed regarding the number of calretinin^+^ cells between all groups at 4 and 8 days. **(C)** An upregulation of the relative *CALRETININ* mRNA expression was detected in filter retinas compared to control samples at 4 days (*p* = 0.001). No differences were noted at 8 days. **(D)** The number of bipolar cells was not altered in any group at 4 days. However, fewer PKCα^+^ cells were discovered in control (*p* = 0.04), filter (*p* = 0.02), and tweezers retinas (*p* = 0.02) compared to those in native samples at 8 days. **(E)** A significant *PKC*α mRNA downregulation was observed in filter samples compared to control ones at 8 days (*p* = 0.02). ONL, outer nuclear layer; OPL, outer plexiform layer; INL, inner nuclear layer; IPL, inner plexiform layer. Scale bar: 20 μm, values are mean ± SEM for immunofluorescence (IF) and median ± quartile + min/max for quantitative real-time PCR (RT-qPCR). IF: *n* = 9–10/group; RT-qPCR: *n* = 5/group. *p < 0.05 vs. native group; ^#^*p* < 0.05 and ^##^*p* < 0.01 vs. controls.

Characteristic cell types of the inner nuclear layer are amacrine and bipolar cells, which were analyzed to investigate the integrity of the inner retina layer (Figure 5A). No difference in the number of calretinin^+^ cells was detected in controls in comparison to native samples (*p* = 0.93; Figure 5B). Also, with the novel techniques, namely, filter (*p* = 0.62) and tweezers (*p* = 1.00), a similar cell number as in native samples was noted at 4 days of cultivation. The same was the case when comparing the filter (*p* = 0.93) and tweezers method (*p* = 0.96) to the controls. Furthermore, after 8 days of cultivation, a slightly lower number of calretinin^+^ cells was observed in all three groups compared to the native situation; however, this cell loss was not significant (control: *p* = 0.10; filter: *p* = 0.07; tweezers: *p* = 0.07). In addition, no significant differences were detected between filter (*p* = 1.00) or tweezers samples (*p* = 1.00) and controls.

To quantify *CALRETININ* on the mRNA level, RT-qPCR analysis was performed (Figure 5C). An upregulation of relative *CALRETININ* mRNA expression was detected in filter retinas (2.1-fold, *p* = 0.001) in comparison to control samples at 4 days of cultivation. The expression in tweezers samples was similar to controls (0.1-fold, *p* = 0.93). Interestingly, at 8 days of cultivation, no difference was measured neither in the filter (0.4-fold, *p* = 0.09) nor in the tweezers group (0.7-fold, *p* = 0.28) in comparison to control retinas.

Bipolar cells in the inner nuclear layer were examined using PKCα labeling. When comparing control retinas to native samples, no difference in cell numbers was found (*p* = 0.76; Figure 5D). With both novel techniques, the amount of PKCα^+^ cells also remained nearly unchanged when compared to native (filter: *p* = 0.98; tweezers: *p* = 0.65) and control samples (filter: *p* = 0.94; tweezers: *p* = 1.0). Notably, the number of PKCα^+^ cells decreased significantly in all three groups at 8 days of cultivation compared to native samples (control: *p* = 0.04; filter: *p* = 0.02; tweezers: *p* = 0.02). In contrast, no alterations in PKCα^+^ cell counts were seen in filter (*p* = 1.00) and tweezers retinas (*p* = 1.00) compared to the controls.

The RT-qPCR examination of *PKC*α mRNA expression revealed no alteration in filter samples compared to the controls (1.7-fold, *p* = 0.31; Figure 5E). Likewise, retina samples cultivated for 4 days *via* the tweezers method showed no significant difference in the *PKC*α expression compared to control samples (0.8-fold, *p* = 0.64). A significant downregulation was also visible in *PKC*α mRNA expression of filter samples compared to the controls after 8 days of cultivation (0.3-fold, *p* = 0.02). In contrast, no alteration was detectable in the mRNA expression of *PKC*α in tweezers retinas compared to the controls (1.0-fold, *p* = 0.99).

### Loss of Retinal Ganglion Cells in Novel Explant Methods

**FIGURE 6:**
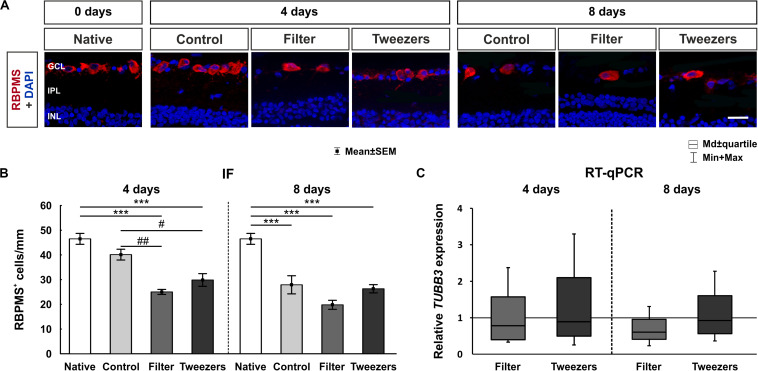
Evaluation of retinal ganglion cells (RGCs). **(A)** RGCs were labeled with RNA-binding protein with multiple splicing (RBPMS) (red) and cell nuclei with 4′,6-diamidino-2-phenylindole (DAPI) (blue). **(B)** The amount of RBPMS^+^ cells in control retinas was comparable to that in native ones. The number of RGCs was significantly reduced in both novel methods, filter (*p* < 0.001) and tweezers (*p* < 0.001), compared to the native method at 4 days. A significant loss of RGCs was noted in filter (*p* = 0.002) and tweezers samples (*p* = 0.03) compared to control ones. At 8 days, fewer RGCs were visible for all three groups control, filter, and tweezers compared to those in native samples (all: *p* < 0.001). **(C)** No differences in the *TUBB3* mRNA expression levels were detected between all groups at 4 and 8 days. GCL, ganglion cell layer; IPL, inner plexiform layer; INL, inner nuclear layer. Scale bar: 20 μm, values are mean ± SEM for immunofluorescence (IF) and median ± quartile + min/max for quantitative real-time PCR (RT-qPCR). IF: *n* = 9–10/group; RT-qPCR: *n* = 5/group. ***p < 0.001 vs. native group; ^#^*p* < 0.05 and ^##^*p* < 0.01 vs. controls.

To evaluate the effects of the novel cultivation methods on RGCs, they were examined using an anti-RBPMS antibody (Figure 6A). No RGC loss was noted in retinas gained *via* the control technique compared to native samples at 4 days of cultivation (*p* = 0.14; Figure 6B). On the contrary, comparing the novel methods filter (*p* < 0.001) and tweezers (*p* < 0.001) to native retinas, a severe loss of RGCs was seen after 4 days of cultivation. A significantly decreased number of RGCs were observed in filter (*p* = 0.002) and tweezers retinas (*p* = 0.03) compared to the controls. With the ongoing time of cultivation, the RGC loss progressed. At 8 days of cultivation, the number of RGCs was significantly lower in control retinas compared to native explants (*p* < 0.001). A severe decrease in RGC numbers was also noted in samples gained *via* filter (*p* < 0.001) and tweezers method (*p* < 0.001) compared to native retinas. With both novel methods, filter (*p* = 0.16) and tweezers (*p* = 0.97), the number of RGCs was comparable to control retinas at 8 days.

RT-qPCR was used to evaluate β*-III-Tubulin (TUBB3)* gene expression in retina samples of all groups, since this gene is enriched in RGCs (Soto et al., 2008; Jiang et al., 2015). The relative *TUBB3* mRNA expression was neither altered in filter (0.8-fold, *p* = 0.43) nor in tweezers retinas (0.9-fold, *p* = 0.74) compared to retinas gained *via* control technique after 4 days of cultivation (Figure 6C). In retinas of the filter technique, a trend toward a downregulation of *TUBB3* gene expression (0.6-fold, *p* = 0.06) was noted in comparison to control samples at 8 days. However, no changes in mRNA level were found for tweezers retinas (0.9-fold, *p* = 0.78).

## Discussion

AMD is a multifactorial disease and one of the major reasons for irreversible blindness. Although there are animal models and cell culture approaches available, there is a certain demand for organ culture models or organoids, mimicking the molecular mechanisms contributing to AMD. This need also applies to other retinal diseases, such as diabetic retinopathy or *retinitis pigmentosa*. Cell cultures have certain limitations, especially a good photoreceptor cell line does not really exist, and working with primary photoreceptor cells has certain obstacles (MacLeod et al., 1999; Romano and Hicks, 2007). Animal models often only mimic specific aspects of retinal disease, while retinal explant cultures provide a simplified system for investigating the retinal function and possible pathomechanisms of these diseases (Murali et al., 2019; Schnichels et al., 2019). Organ cultures still possess elementary structures of the organ, in this case the retina, allowing analysis of complex interactions, e.g., signaling pathways.

To this end, we evaluated new techniques for the preparation of porcine organotypic neuroretina explants, which should preserve the bacillary layer in a better fashion than previous protocols. The two novel methods, named tweezers and filter, resulted in a better conservation of the sensitive rod and L-cone cells than the control technique. The results demonstrated that *via* rotation of 180°, hence having the photoreceptor layer facing up during cultivation, the retinal morphology could be maintained much better. Therefore, this *ex vivo* model should mimic the *in vivo* situation.

*Ex vivo* cultivation of photoreceptor cells is complicated for several reasons. Many degenerative processes are directly initiated through the explantation of the neuroretina. The detachment of photoreceptor cells from the RPE is inducing rapid apoptotic processes (Cook et al., 1995). Hence, a sensitive method, with as little physical manipulation as possible, is mandatory for the preparation of adult neuroretina explants. Regarding these facts, our methods aimed to explant the retina using a “no touch” technique to minimize the harm to the retina as much as possible. The investigation of the total retina thickness revealed a better maintenance of retinas in the tweezers group compared to control and filter retinas over the cultivation time. Our study suggested that omitting direct physical contact using the two new techniques led to an improved preservation of rods and L-cones. This preservation of photoreceptors could not be noted in the control group, where these cells had direct contact to the insert. This led to a thinning of the whole retina. This effect can be explained by looking closer at the morphology of rods and cones. Compared to other neuronal cell types of the retina, both photoreceptor cell types have a more elongated thin shape of the outer segment, resulting in easy breakage of the sensitive connection to the photoreceptor nuclei (Mustafi et al., 2009; Kawamura and Tachibanaki, 2012).

OCT is an interferometry and non-invasive technique that can be used to acquire cross-sectional tomographic pictures. This enables recording of dynamic changes in the course and progression of diseases. In AMD, the ultrastructure of drusen as well as geographic atrophy can be imaged and characterized (Khanifar et al., 2008; Yehoshua et al., 2010). The advantages of this method can also be applied in animal models or organ cultures. Therefore, in this study, the retinal explants were evaluated by SD-OCT as well as by histology (H&E staining). Interestingly, the results between both methods differed. For example, the filter samples appeared less preserved in SD-OCT than in the H&E staining. In contrast, the tweezers group had a significant thicker total retina *via* SD-OCT measurements, but not after H&E staining at 8 days. This could be explained by the fact that disruption of the retina can be generated during dissection, embedding, cutting, and staining for H&E (Dailey et al., 2017). Especially, processing the samples of the filter group could be worse through the attached filter. The use of the SD-OCT provides the ability to measure the same sample over time, while for H&E analyses, new samples are needed for every evaluation time point. In future studies, SD-OCT measurements could help to identify the development and progression of drusen in an AMD-like coculture system.

In general, a longer cultivation time makes a preservation of retina less likely, which applies to all neuronal cell types. In this aspect, cultivation time should be kept as short as possible but also adequately mimic the *in vivo* situation and give enough time for studies. Therefore, we were interested in adapting and improving the cultivation method of photoreceptor cells to extend the cultivation time and enabling us to analyze aging effects. The rhodopsin and opsin signals in tweezers samples in our study were comparable to those of the native samples at 8 days. Previous studies using mouse and porcine retinas revealed that photoreceptors become pyknotic after 3–4 days *in vitro* (Tansley, 1933; Ogilvie et al., 2000; Taylor et al., 2013a,b). However, Wang et al. (2011) demonstrated that the rotation and inner retina support conserved the photoreceptor layer for up to 7 days in culture. A loss of neuronal cells in an adult explant culture system is given through the limitations of an *ex vivo* culture, such as the detachment of supporting tissue, the missing RPE cells, and the lack of choroidal circulation. In contrast, our explants cultured using the novel techniques (filter and tweezers) displayed a significantly better photoreceptor survival than the control technique. The number of opsin^+^ cells was, even after 8 days *ex vivo*, still comparable to the number of the native samples. Moreover, the rhodopsin signal intensity was well preserved in tweezers samples and comparable to native samples after 4 days of cultivation. Also, *OPSIN* and *RHODOPSIN* mRNA expression in the tweezers group was upregulated after 8 days of cultivation, which indicates a preserved photoreceptor cell health. However, the opsin^+^ cells appeared more disorganized compared to native samples at this time point. This may influence the function of these cells. In future studies, electroretinography should be included to clarify this point.

To investigate the effects of the different methods on the inner retina, amacrine and bipolar cells were analyzed. Interestingly, the number of calretinin^+^ cells was not altered in all groups. In contrast, an upregulation of *CALRETININ* mRNA was found in the filter group at 4 days. The used antibody against PKCα is specific for rod bipolar cells, which are representing only a part of the bipolar cells of the retina. The amount of PKCα^+^ cells was stable in explants of all techniques at 4 days. However, at day 8, a significant loss of bipolar cells was visible in all three techniques compared to native controls. Thus, a progressive loss of PKCα^+^ cells was detectable with ongoing cultivation. This result was supported by a significant downregulation of the *PKC*α mRNA expression in filter samples cultivated for 8 days. Consequently, our findings indicate that in neuroretina explant cultures, bipolar cells are probably more sensitive than amacrine cells. Amacrine cells represent a very diverse class of intrinsic interneurons in the inner retina, forming a network. Hence, they receive synaptic input from other amacrine cells as well as bipolar cells. They provide this input to further amacrine cells, bipolar cells, and RGCs (Wilson and Vaney, 2010). Bipolar cells interact directly with RGCs or indirectly through the amacrine cells (Fitzpatrick, 2015). Stained amacrine and bipolar cell types are located in the inner retina, but they are affected differently. Interestingly, Fernandez-Bueno et al. (2012) made the same observation when cultivating human retinas. They discovered a loss of bipolar cells and impairment of their axons with ongoing time of cultivation, while such a degenerating process was not documented for amacrine cells (Fernandez-Bueno et al., 2012). The loss of RGCs in our study was severe in filter and tweezers samples already at 4 days of cultivation and increased over time. These results confirm that direct contact with the membrane fosters cell damage. The RGCs are axotomized and hence deprived of their trophic support, resulting in apoptosis. The bipolar cells are connected to the RGCs, so an increased degeneration at 8 days of cultivation might indirectly also affect them.

The aim of this study was to find a suitable preparation technique that preserves photoreceptor cells in a porcine organ culture model. This was successfully achieved by introducing the tweezers and filter method. Both new methods led to a significantly improved morphology of photoreceptors, making them more comparable to the *in vivo* situation. Although both methods revealed just small differences in comparison, the tweezers method showed more preserved photoreceptor cells (protein and mRNA level) and a better morphology *via* SD-OCT. In addition, handling of the explants was much easier with the tweezers method, leading to a higher reproducibility. Consequently, this method seems to be more adequate for following coculture experiments of RPE and neuroretina. The improved photoreceptor cultivation should enable us to analyze the interaction of RPE and photoreceptor cells. Both structures and their interaction are essential in understanding the pathomechanisms underlying AMD. To reproduce an AMD-like pathology, RPE and a functional barrier are needed to induce drusen. Pilgrim et al. (2017) already revealed that in a primary RPE cell culture system, sub-RPE deposits were formed. These deposits contained, for example, proteins, lipids, and hydroxyapatite, as seen in AMD patients (Pilgrim et al., 2017). These drusen-like deposits should also be implemented in future coculture models for AMD research.

In conclusion, this work provides two explant methods for organotypic porcine retina culture models focusing on photoreceptors. Both novel methods improve photoreceptor cultivation in contrast to the established control technique. Especially, the tweezers method facilitates the analysis of photoreceptor degeneration and can be further utilized to study different diseases, such as AMD, diabetic retinopathy, or *retinitis pigmentosa*. Furthermore, the tweezers method could be used in a coculture system of neuroretina and RPE cells, which would provide a promising and innovative technique to effectively reduce the number of animal experiments in retina research.

## Data Availability Statement

The raw data supporting the conclusions of this article will be made available by the authors, without undue reservation.

## Author Contributions

NW performed the experiments, analyzed the data, and wrote the manuscript. SR performed the experiments and revised the manuscript. MG, AG, and JH performed the experiments and analyzed the data. HD revised the manuscript. SJ and SS designed the study and revised the manuscript. All authors read and approved the final version of the manuscript.

## Conflict of Interest

The authors declare that the research was conducted in the absence of any commercial or financial relationships that could be construed as a potential conflict of interest.

## Footnotes

1 http://www.bioinformatics.nl/cgi-bin/primer3plus/primer3plus.cgi/

## Supplementary Material

Supplementary Figure 1 Comparison of the three methods on day 0 *via* SD-OCT and H&E staining. **(A)** Exemplary SD-OCT pictures of all used techniques at day 0. **(B)** No difference in the total retina thickness was observed between all three techniques at day 0 (= native). The filter (*p* = 1.00) as well as the tweezers samples (*p* = 0.76) were comparable to the control ones. Furthermore, no alterations were noted between tweezers and filter native retinas (*p* = 0.76). **(C)** All samples were stained with H&E. No difference in the morphology or structure was noted in retinas from all three explantation techniques. **(D)** The statistical evaluation showed no difference in the total retinal thickness (all: *p* > 0.05). OS, photoreceptor outer segments; ONL, outer nuclear layer; OPL, outer plexiform layer; INL, inner nuclear layer; IPL, inner plexiform layer; GCL, ganglion cell layer. Scale bar: 50 μm, values are mean ± SEM. *n* = 9–10/group.
