# Supplementary figures and images for: Novel Porcine Retina Cultivation Techniques Provide Improved Photoreceptor Preservation
Source: Front Neurosci. 2020 Oct 6;14:556700. doi: 10.3389/fnins.2020.556700 (PMC7573241; doi:10.3389/fnins.2020.556700)

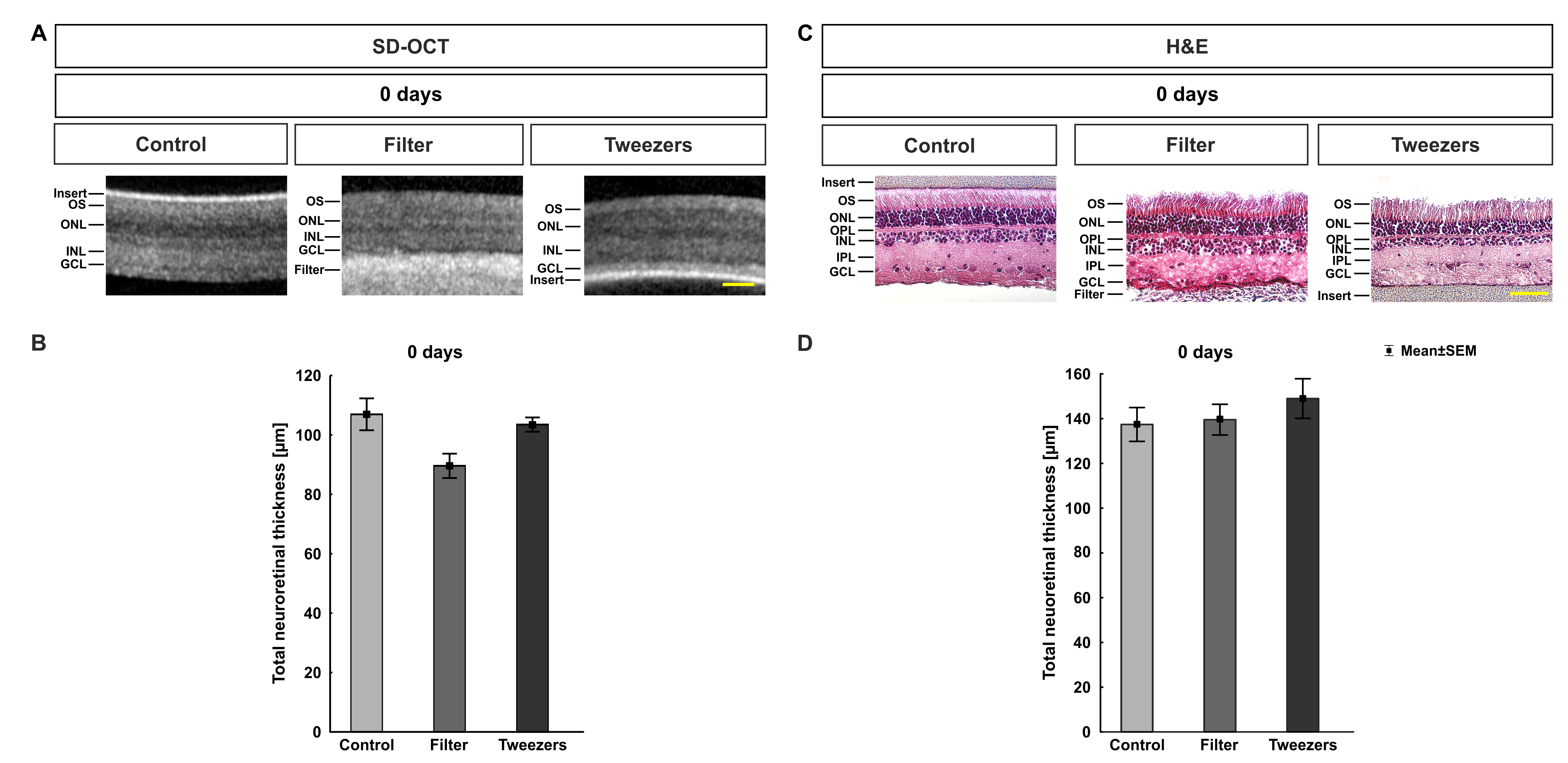

Supplement: Supplementary Figure 1 — Comparison of the three methods on day 0 via SD-OCT and H&E staining. (A) Exemplary SD-OCT pictures of all used techniques at day 0. (B) No difference in the total retina thickness was observed between all three techniques at day 0 (= native). The filter (p = 1.00) as well as the tweezers samples (p = 0.76) were comparable to the control ones. Furthermore, no alterations were noted between tweezers and filter native retinas (p = 0.76). (C) All samples were stained with H&E. No difference in the morphology or structure was noted in retinas from all three explantation techniques. (D) The statistical evaluation showed no difference in the total retinal thickness (all: p > 0.05). OS, photoreceptor outer segments; ONL, outer nuclear layer; OPL, outer plexiform layer; INL, inner nuclear layer; IPL, inner plexiform layer; GCL, ganglion cell layer. Scale bar: 50 μm, values are mean ± SEM. n = 9–10/group. [file Image_1.tif]
